# Defective Lipid Droplet–Lysosome Interaction Causes Fatty Liver Disease as Evidenced by Human Mutations in TMEM199 and CCDC115

**DOI:** 10.1016/j.jcmgh.2021.09.013

**Published:** 2021-10-07

**Authors:** Lars E. Larsen, Marjolein A.W. van den Boogert, Wilson A. Rios-Ocampo, Jos C. Jansen, Donna Conlon, Patrick L.E. Chong, J. Han M. Levels, Roos E. Eilers, Vinay V. Sachdev, Noam Zelcer, Tobias Raabe, Miao He, Nicholas J. Hand, Joost P.H. Drenth, David J. Rader, Eric S.G. Stroes, Dirk J. Lefeber, Johan W. Jonker, Adriaan G. Holleboom

**Affiliations:** 1 Department of Vascular Medicine, Amsterdam UMC, location AMC, Amsterdam, The Netherlands; 2 Department of Pediatrics, Section Molecular Metabolism and Nutrition, University Medical Center Groningen, University of Groningen, The Netherlands; 3 Department of Gastroenterology and Hepatology, Radboud University Medical Center, Nijmegen, The Netherlands; 4 Department of Medicine, Perelman School of Medicine at the University of Pennsylvania, Philadelphia, Pennsylvania; 5 Department of Medical Biochemistry, Amsterdam University Medical Centers, location AMC, Amsterdam, The Netherlands; 6 Department of Pathology and Laboratory Medicine, Perelman School of Medicine at the University of Pennsylvania, Philadelphia, Pennsylvania; 7 Division of Laboratory Medicine, The Children’s Hospital of Philadelphia, Philadelphia, Pennsylvania; 8 Department of Genetics, Perelman School of Medicine at the University of Pennsylvania, Philadelphia, Pennsylvania; 9 Department of Laboratory Medicine, Translational Metabolic Laboratory, Radboud University Medical Center, Nijmegen, The Netherlands

**Keywords:** V-ATPase assembly defects, Lipophagy, Lipid droplet, Fatty liver disease, NAFLD, Hyperlipidemia, Mutations in TMEM199 and in CCDC115, apoB, apolipoprotein B, BSA, bovine serum albumin, DMEM, Dulbecco modified Eagle medium, FPLC, fast protein liquid chromatography, HDL-c, high-density lipoprotein-cholesterol, HLC, hepatocyte-like cell, iPSc, induced pluripotent stem cell, LAL, lysosomal lipase, LDL-c, low-density lipoprotein-cholesterol, MALDI-TOF, matrix-associated laser desorption/ionization time-of-flight, NAFLD, nonalcoholic fatty liver disease, OA, oleic acid, qPCR, quantitative polymerase chain reaction, siRNA, small interfering RNA, TC, total cholesterol, TG, triglyceride, VLDL, very low density lipoprotein, V-ATPase, vacuolar-type H^+^-adenosine triphosphatase

## Abstract

**Background & Aims:**

Recently, novel inborn errors of metabolism were identified because of mutations in V-ATPase assembly factors *TMEM199* and *CCDC115*. Patients are characterized by generalized protein glycosylation defects, hypercholesterolemia, and fatty liver disease. Here, we set out to characterize the lipid and fatty liver phenotype in human plasma, cell models, and a mouse model.

**Methods and Results:**

Patients with *TMEM199* and *CCDC115* mutations displayed hyperlipidemia, characterized by increased levels of lipoproteins in the very low density lipoprotein range. HepG2 hepatoma cells, in which the expression of *TMEM199* and *CCDC115* was silenced, and induced pluripotent stem cell (iPSC)-derived hepatocyte-like cells from patients with *TMEM199* mutations showed markedly increased secretion of apolipoprotein B (apoB) compared with controls. A mouse model for TMEM199 deficiency with a CRISPR/Cas9-mediated knock-in of the human A7E mutation had marked hepatic steatosis on chow diet. Plasma N-glycans were hypogalactosylated, consistent with the patient phenotype, but no clear plasma lipid abnormalities were observed in the mouse model. In the *siTMEM199* and *siCCDC115* HepG2 hepatocyte models, increased numbers and size of lipid droplets were observed, including abnormally large lipid droplets, which colocalized with lysosomes. Excessive de novo lipogenesis, failing oxidative capacity, and elevated lipid uptake were not observed. Further investigation of lysosomal function revealed impaired acidification combined with impaired autophagic capacity.

**Conclusions:**

Our data suggest that the hypercholesterolemia in *TMEM199* and *CCDC115* deficiency is due to increased secretion of apoB-containing particles. This may in turn be secondary to the hepatic steatosis observed in these patients as well as in the mouse model. Mechanistically, we observed impaired lysosomal function characterized by reduced acidification, autophagy, and increased lysosomal lipid accumulation. These findings could explain the hepatic steatosis seen in patients and highlight the importance of lipophagy in fatty liver disease. Because this pathway remains understudied and its regulation is largely untargeted, further exploration of this pathway may offer novel strategies for therapeutic interventions to reduce lipotoxicity in fatty liver disease.

## Graphical abstract

**Figure:**
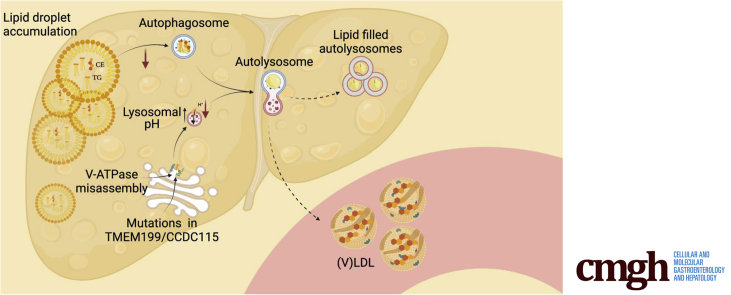

**Summary**

TMEM199 and CCDC115 deficiency are V-ATPase assembly defects, characterized by fatty liver disease and hyperlipidemia. Liver cell models and a mouse model demonstrated increased lysosomal lipid accumulation, impaired lysosomal acidification, and impaired autophagic capacity, highlighting the importance of lipophagy in fatty liver disease.

The multi-subunit vacuolar-type H^+^-ATPase (V-ATPase) is responsible for acidification of intracellular organelles, thereby controlling several pH-sensitive intracellular processes such as protein glycosylation, lysosomal lipase (LAL) activity, and autophagy.^1^ By sequencing human homologues of yeast V-ATPase assembly factors in families with formerly unsolved N- and mucin-type O-glycosylation abnormalities, 2 novel inborn errors of metabolism were identified with defects in 2 of these assembly factors, TMEM199 and CCDC115.^2^^,^^3^ Interestingly, affected patients displayed fatty liver disease and marked hypercholesterolemia but did not exhibit mutations in any of the genes known to cause familial hypercholesterolemia (ie, *LDLR, APOB,* or *PCSK9*) or mutations in *LIPA*, encoding LAL, which is associated with cholesterol ester storage disease.

Four of the patients described had mutations in *TMEM199,* the human homologue of yeast V-ATPase assembly factor Vma12p. They presented with hepatic steatosis, elevated transaminases, mild fibrosis, and hypercholesterolemia. Their serum glycosylation pattern was similar to that seen in other mixed glycosylation defects, specifically N-glycans lacking galactose and sialic acids and abnormal mucin-type O-glycosylation of apolipoprotein C-III.^2^

In 3 other unrelated families, we found mutations in another V-ATPase assembly factor, *CCDC115*, the human homologue of yeast Vma22p. Affected individuals showed marked hypercholesterolemia and a storage disease-like phenotype with hepatosplenomegaly and steatohepatitis with fibrosis. In 2 patients, this led to liver failure requiring transplantation. Serum glycosylation analysis revealed abnormal N- and O-glycosylation, which was confirmed in patient fibroblasts and could be rescued by lentiviral expression of type CCDC115.^3^

Notably, overlapping liver and lipid phenotypes have been described in patients with mutations in other assembly factors of the V-ATPase as well, ie, *ATP6AP1,*^4^ *ATP6AP2,*^5^ and *VMA21.*^6^ The phenotypes of those patients range from mild steatohepatitis with mildly elevated liver transaminases to early cirrhosis and liver failure. In addition, they had similar defects in N- and O-glycosylation, associated with impaired Golgi homeostasis. Together, this suggests that V-ATPase misassembly is the common pathogenic process underlying the liver and lipid phenotypes observed in these syndromes.

Here, we characterized the mechanisms underlying the hypercholesterolemia and fatty liver phenotypes caused by *TMEM199* and *CCDC115* deficiency. Our results from 2 hepatocyte models suggest defective lipid droplet–lysosome interaction as an underlying mechanism for the lipotoxic hepatic phenotype in these V-ATPase assembly defects. In addition, we confirmed the observed fatty liver phenotype in a murine model of *TMEM199* deficiency. The hyperlipidemic phenotype could be explained by increased secretion of apolipoprotein B (apoB), most likely as a result of the steatosis, which was observed in both *TMEM199* and *CCDC115* knockdown cell lines as well as in the *TMEM199* knockout mouse model. Together, our results point toward defective lipid droplet–lysosome interaction as an underlying mechanism for the lipotoxic hepatic phenotype in these V-ATPase assembly defects. These findings bear relevance for the understudied process of lipophagy in nonalcoholic fatty liver disease (NAFLD), the rapidly increasing common form of fatty liver disease associated with obesity and type 2 diabetes mellitus.

## Results

### *Patients With* TMEM199 *and* CCDC115 *Mutations Have Hypercholesterolemia and Increased ApoB Secretion*

**Figure 1:**
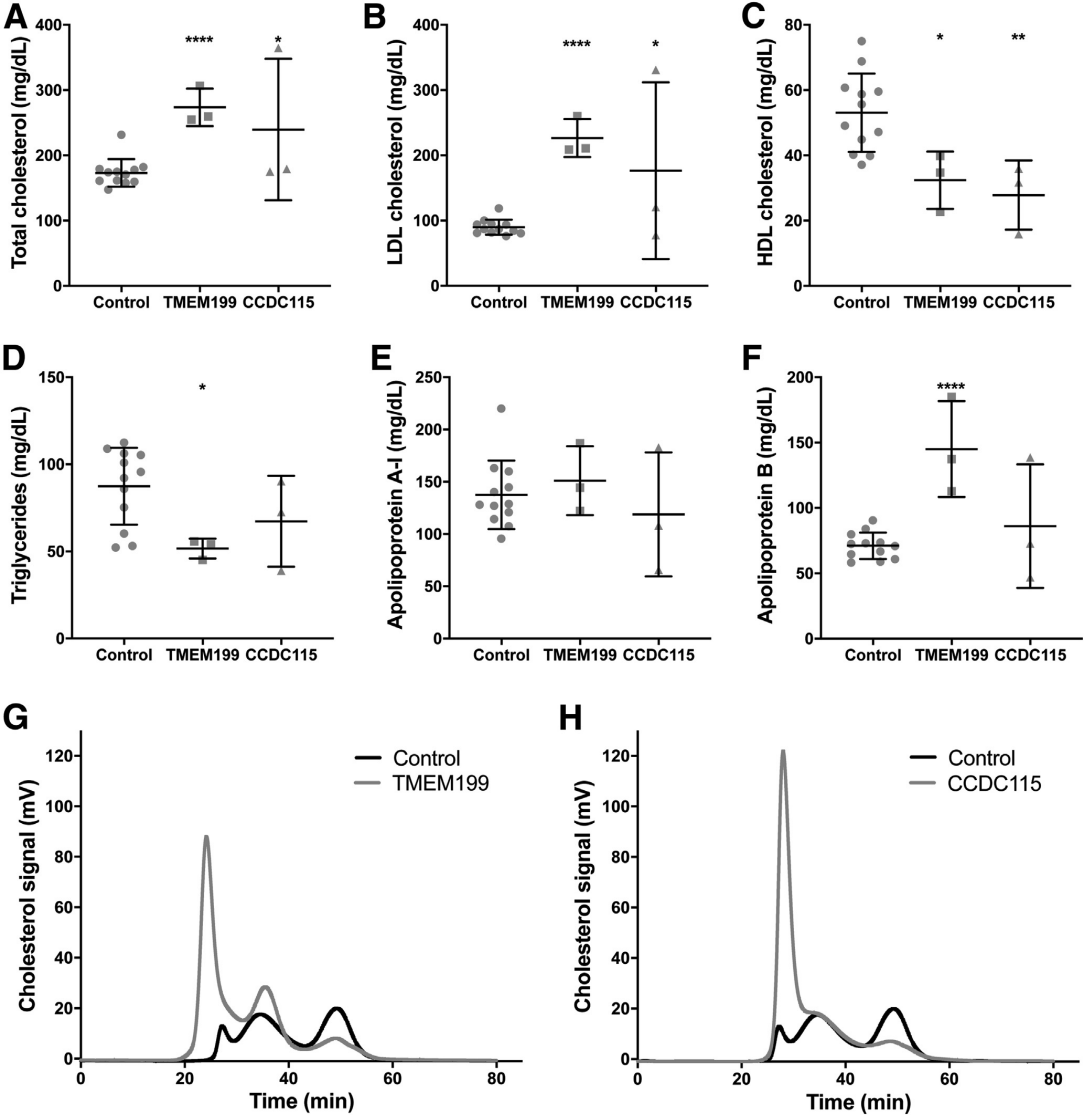
***TMEM199*- and *CCDC115-CDG* patients have hypercholesterolemia.** Plasma levels of (*A*) TC, (*B*) LDL-c, (*C*) HDL-c, (*D*) TG, (*E*) apoA-I, and (*F*) apoB in 3 *TMEM199-* and 3 *CCDC115-CDG* patients after overnight fast and compared with 12 age- and gender-matched controls. Representative traces of FPLC of (*G*) 1 *TMEM199* patient and (*H*) 1 *CCDC115-CDG* patient are shown with the trace of pooled control plasma. All measurements are shown as a scatter plot with mean ± standard deviation, and each data point depicts a single measure from an individual subject plasma sample.

Plasma lipids were measured in 3 patients with *TMEM199* mutations, 3 with *CCDC115* mutations, and in 12 age- and gender-matched controls. The results are shown in Figure 1. The average age of the *TMEM199* patients was 10 ± 1 years and similar to controls (10 ± 2 years; *P* = .852). The *CCDC115* patients were much younger, with average age of 4 ± 4 years (*P* = .009). There was more than 1.5-fold increase in total cholesterol (TC) in the *TMEM199* and *CCDC115* patients (274 ± 29 and 240 ± 108 mg/dL, respectively) compared with controls (173 ± 21 mg/dL; *P* < .0001 and *P* = .046, respectively; Figure 1*A*). Low-density lipoprotein-cholesterol (LDL-c) was increased approximately 2-fold in patients (227 ± 29 and 177 ± 136 mg/dL) compared with controls (90 ± 12 mg/dL; *P* < .0001 and *P* = .028, respectively; Figure 1*B*). Plasma levels of ApoB were also significantly increased in *TMEM199* patients (119 ± 77 mg/dL) compared with controls (71 ± 10 mg/dL; *P* < .0001; Figure 1*F*), but this was not observed in *CCDC115* patients. Interestingly, fast protein liquid chromatography (FPLC) analysis of plasma cholesterol showed an altered cholesterol distribution in patients with *TMEM199* and *CCDC115* mutations, with considerable increase of the cholesterol content in large very low density lipoprotein (VLDL) particles (Figure 1*G* and *H*).

High-density lipoprotein-cholesterol (HDL-c) was significantly lower in both *TMEM199* and *CCDC115* patients (32 ± 9 and 28 ± 11 mg/dL) versus controls (53 ± 12 mg/dL; *P* = .016 and .006, respectively; Figure 1*C*). Triglyceride (TG) was significantly lower in *TMEM199* patients (52 ± 6 mg/dL) but comparable in *CCDC115* patients (67 ± 26 mg/dL) with controls (87 ± 22 mg/dL; *P* = .018 and *P* = .194, respectively). ApoA1 was not different between patients and controls (151 ± 33 and 119 ± 59 mg/dL versus 138 ± 33 mg/dL in controls; *P* = .532 and *P* = .461, respectively; Figure 1*E*).

### *Cellular Models of* TMEM199 *and* CCDC115 *Deficiency Display Increased ApoB Secretion*

**Figure 2:**
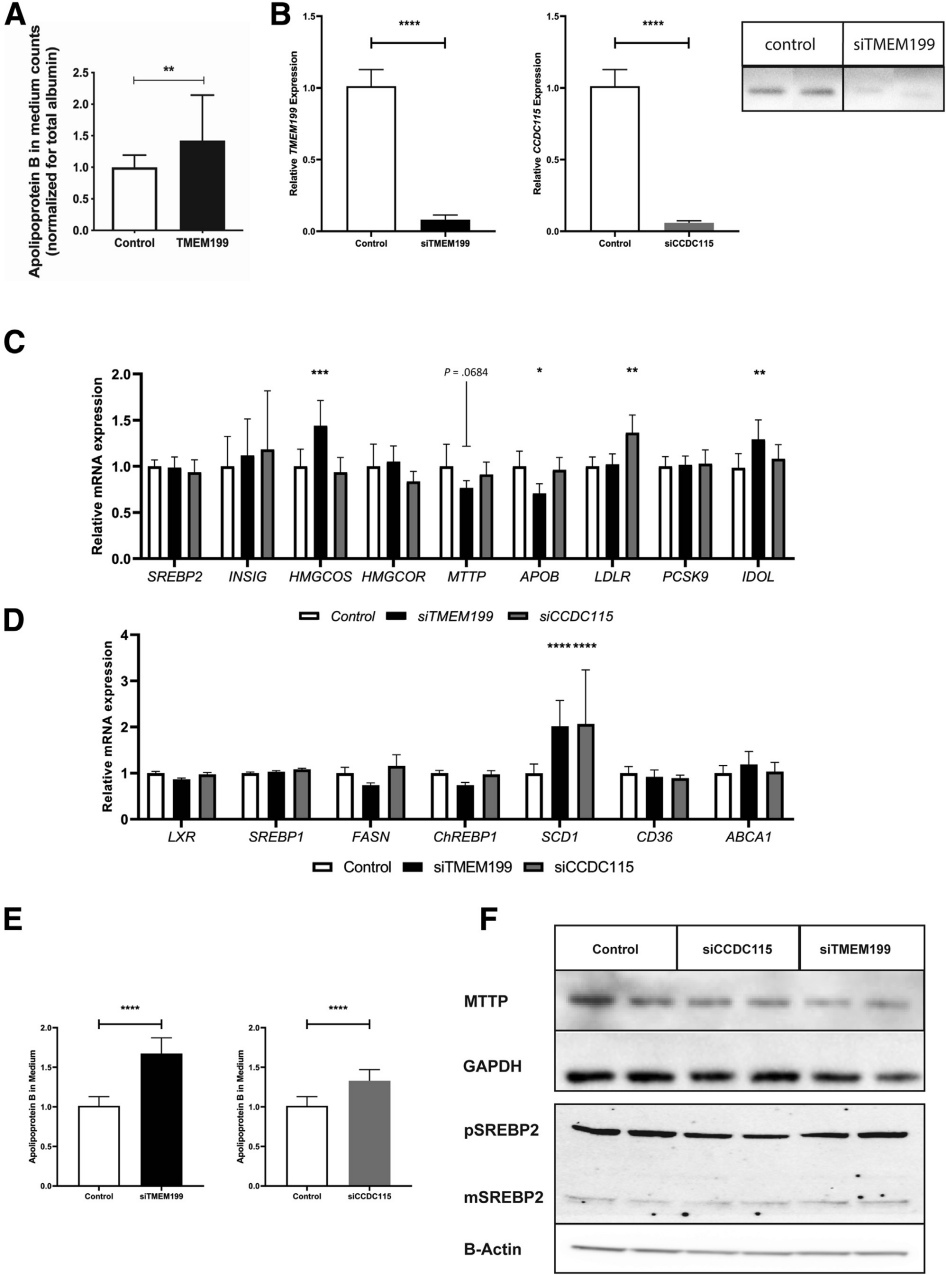
**ApoB secretion in *TMEM199*-deficient HLCs and *siTMEM199* and *siCCDC115* treated HepG2 cells.** (*A*) ApoB secretion in TMEM199-deficient HLCs. Panel shows relative ^35^S apoB secretion in medium of patient-derived HLCs. (*B*) TMEM199 and CCDC115 mRNA expression levels *(left)* and TMEM199 protein levels *(right)* in siTMEM199 and siCCDC115 treated HepG2 cells, (*C*) mRNA expression of genes involved in cholesterol metabolism, and (*D*) mRNA expression of genes involved in de novo lipogenesis. (*E*) Secretion of apoB into the medium (normalized for total protein) in OA stimulated conditions for siTMEM199 treated cells *(left)* and siCCDC115 treated cells *(right)*. (*F*) Western blot panel for MTTP (normalized for GAPDH), pre (p)SREBP2 and mature (m)SREBP2 (normalized for B-actin). All panels show representative Western blots or means ± standard deviation from 3 experiments with triplicate measurements per experiment. ∗∗∗∗*P* < .0001, ∗∗∗*P* < .001, ∗*P* < .05, and ns indicates non-significant results as calculated with Student unpaired two-sided *t* tests.

To investigate the observed hypercholesterolemia, we assessed apoB secretion by ^35^S steady-state protein labelling in *TMEM199*-deficient patient-derived hepatocyte-like cells (HLCs) and found significantly increased apoB secretion (42% increase; *P* = .008; Figure 2*A*) as compared with controls. Next, we studied apoB secretion in HepG2 cells in which either *TMEM199* or *CCDC115* was silenced by using small interfering RNAs (siRNAs), using non-targeting siRNAs as control. We achieved effective silencing of 90% and 93% for *TMEM199* and *CCDC115,* respectively, and resulting in strongly reduced protein expression of TMEM199 (no antibody for Western blot available for CCDC115*)*; Figure 2*B*). In these cells, we determined the expression of key genes in lipid metabolism by using quantitative polymerase chain reaction (qPCR) (Figure 2*C* and *D*). Compared with controls, there was no significant difference in expression of *APOB, MTTP,* or *SREBP2* in both *siTMEM199* and *siCCDC115* treated cells. However, there was increased expression of the lipid synthesis gene *SCD1* in both *siTMEM199* and *siCCDC115* cells (Figure 2*D*). ApoB secretion was measured with enzyme-linked immunosorbent assay after oleic acid (OA) stimulation and was significantly increased in both *siTMEM199* and *siCCDC115* treated cells compared with controls (75% and 28% increase, respectively; *P* < .0001 and *P* = .01; Figure 2*E*). Finally, protein levels of MTTP and SREBP2 (pre and mature) were not affected in *siTMEM199* and *siCCDC115* treated cells (Figure 2*F*).

### TMEM199*-deficient Mice Display Increased Hepatic Steatosis*

To investigate the mechanism underlying the plasma lipid and fatty liver phenotype we observed in patients, we set out to generate a murine model. Full CRISPR-KO of *Tmem199* turned out to be lethal because there were no viable pups recovered. Therefore, we generated mice carrying the human Ala7Glu mutation, resulting in hypomorphic expression of Tmem199. Homozygous *Tmem199-Ala7Glu* mice did not show reduced embryonic viability as indicated by a normal Mendelian distribution of the 3 genotypes. Homozygotes also did not show any gross abnormalities, and juvenile development progressed comparable with control littermates (wild-type or heterozygous for the *Tmem199-Ala7Glu* allele) with normal weight gain. Similarly, the mice had apparently normal neurologic development and no apparent neuromotor disabilities, as observed in patients (data not shown).

**Figure 3:**
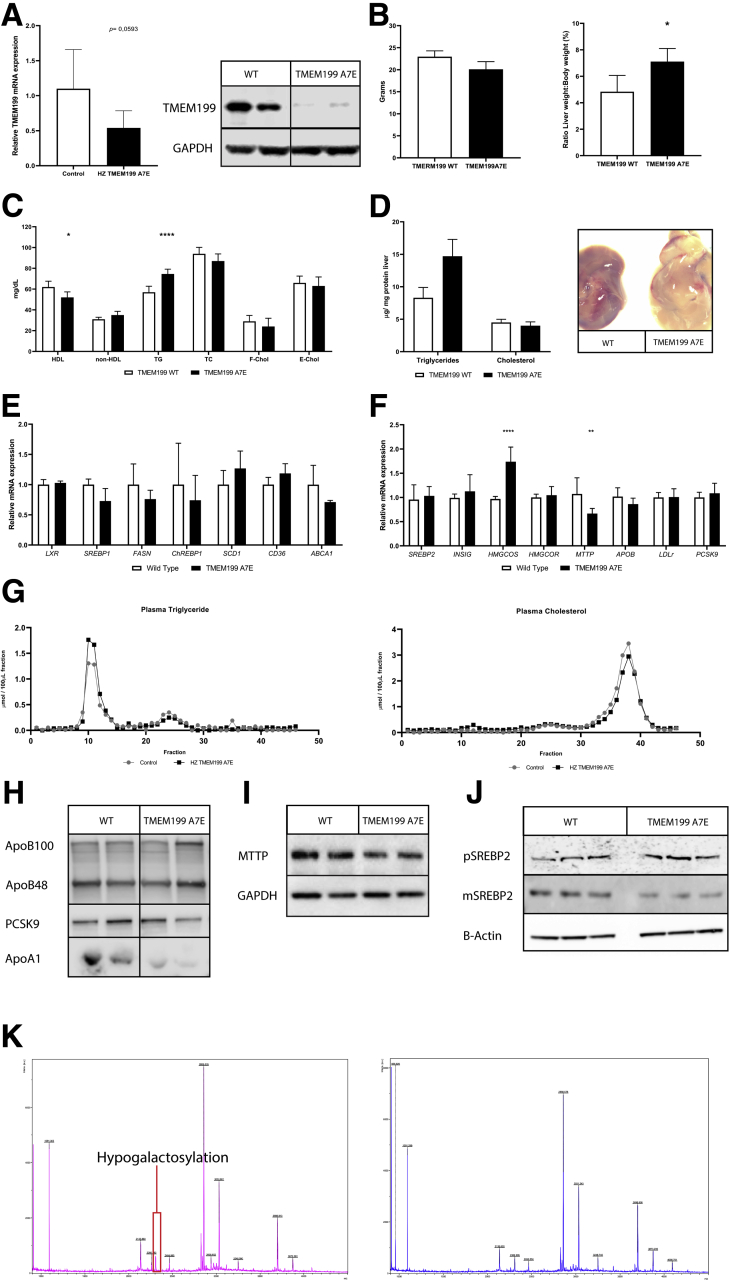
**TMEM199 Ala7Glu mouse model on chow diet.** Liver and plasma of 8-week-old mice were collected in liquid nitrogen or heparin coated tubes, respectively, after 4-hour fast. (*A*) QPCR and Western blot for hepatic *TMEM199* mRNA (*left*) and protein expression (*right*). (*B*) Body weight and liver weight to body weight ratio; liver to body weight ratio was significantly increased. (*C*) Biochemical plasma lipid panel; reduced HDL and increased plasma TG were observed. (*D*) TMEM199-Ala7Glu mice have increased hepatic TG (*left*) and visible hepatic steatosis (*right*). (*E* and *F*) mRNA expression for genes involved in lipid metabolism in mouse livers. (*G*) Plasma of control and heterozygous *Tmem199-Ala7Glu* mice were pooled for FPLC analysis (n = 5 per group; littermates), and fractions were analyzed for TG and cholesterol content. (*H*) ApoB100/48, PCSK9, and apoA1 were analyzed from 7 μL whole plasma on Western blot. (*I* and *J*) MTTP and SREBP2, respectively, on Western blots of mouse liver lysates. All panels show representative Western blots from 3 experiments with triplicate measurements per experiment. ∗∗∗*P* < .001, ∗∗*P* < .01, ∗*P* < .05 as calculated with unpaired Mann-Whitney test. (*K*) N-glycan MALDI-TOF profiles derived from mouse plasma.

For characterization, hepatic *Tmem199* mRNA and protein expression were assessed in 8-week-old mice (n = 5 per group) on chow diet. Relative *Tmem199* mRNA expression showed a reduction of 52% (0.48 ± 0.24 in *Tmem199-Ala7Glu* mice versus 1.00 ± 0.59 in controls; *P* = .059; Figure 3*A*, left panel). Using Western analysis, virtually no Tmem199 protein could be detected in mouse livers homozygous for the *Tmem199-Ala7Glu* mutation (Figure 3*A*, right panel). The mRNA expression of lipid genes in homozygous *Tmem199-Ala7Glu* mice did not reveal any significant changes in either de novo lipogenesis or cholesterol metabolism genes except for HMGCS, which was increased (Figure 3*E* and *F*).

To determine the composition of lipoproteins, heparin-treated plasma was pooled per genotype and analyzed by FPLC. Slightly higher levels of TG were observed in the VLDL-containing fractions of the mutant mice (area under the curve was 4.956 in *Tmem199-Ala7Glu* mutant mice versus 4.012 in controls; Figure 3*G*). Consistent with our observation in the patients, cholesterol content was slightly decreased in the HDL-containing fractions, (area under the curve was 13.50 in mutants versus 15.85 in controls; Figure 3*G*). These differences in TG and HDL were also observed with direct measurements in plasma (Figure 3*C*). Further analysis with Western blot showed that the ratios of apo B100/48 and Pcsk9 levels in plasma were not affected, whereas apoA1 was reduced (Figure 3*H*). No differences in hepatic MTTP or SREBP2 processing were observed by Western blot (Figure 3*I* and *J*).

Consistent with the hepatic phenotype seen in patients with *TMEM199* deficiency, the livers of mice homozygous for *Tmem199-Ala7Glu* clearly were steatotic as indicated by increased levels of hepatic TG (80% higher than in controls, *P* < .01; Figure 3*D*). The mice showed no atherosclerotic lesions in the aortic root at 20 weeks (data not shown). Last, we analyzed mouse plasma N-glycan profiles by matrix-associated laser desorption/ionization time-of-flight (MALDI-TOF) mass spectrometry and found that mice bearing the *Tmem199-Ala7Glu* mutation had 1.5- to 2-fold decrease in complex fucosylated glycans and decreased levels of sialylated and galactosylated glycans as compared with controls (Figure 3*K*, Table 1). This is consistent with the abnormal N-glycan profiles found in *TMEM199*-deficient patients.^2^

**Table 1:** Quantified N-Glycan MALDI-TOF Profiles Derived From Mouse Plasma

					
Wild-type	42%	19%	15%	4%	0%
TMEM199 Ala7Glu	45%	19%	10%	2%	1%

### *Silencing of* TMEM199 *and* CCDC115 *Induces Lipid Accumulation*

**Figure 4:**
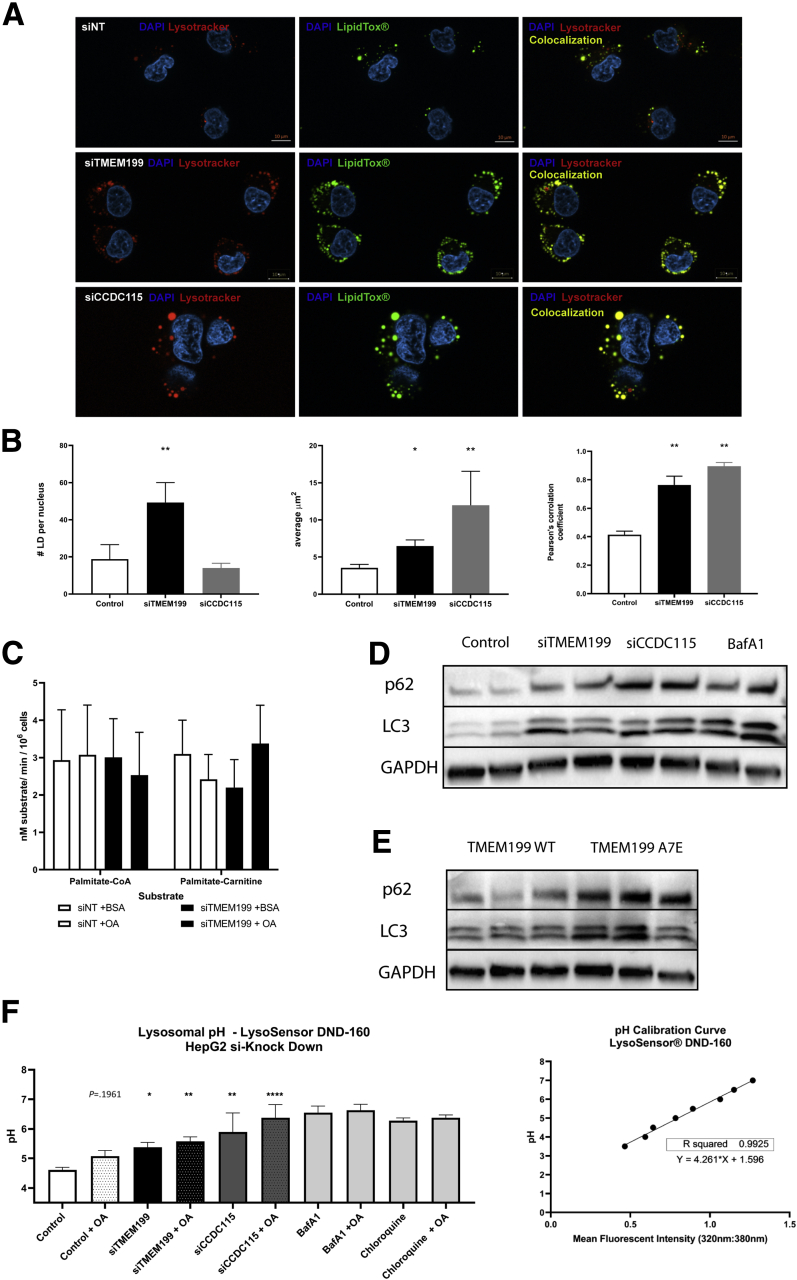
**Lipid droplet accumulation and autophagy analysis of *siTMEM199* and *siCCDC15* HepG2 cells.** HepG2 cells were treated with siRNAs for non-targeted (control), *TMEM199*, and *CCDC115*. (*A*) After 24 hours of siRNA treatment cells were incubated with 200 μmol BSA conjugated OA in full medium for 24 hours. After incubation with OA cellular neutral lipids and lysosomes were labeled with LipidTox and Lysotracker red DND-99, respectively. (*B*) Quantification of lipid droplet size, number of lipids per nucleus, and Pearson’s correlation coefficient for LipidTox Green and Lysotracker Red DND-99. (*C*) Oroboros respiratory capacity in *siTMEM199* HepG2 cells compared with controls. (*D*) HepG2 cells were treated 48 hours with siRNA; Bafilomycin A1 is positive control for V-ATPase mediated induced autophagy inhibition. Panels represent whole cell lysate protein levels of autophagy markers (P62; LC3B) and GAPDH as loading control. (*E*) Mouse liver lysates analyzed for autophagy marker (p62/LC3). (*F*) siRNA treated HepG2 cells were analyzed for lysosomal acidification by LysoSensor DND-160; shown are the pH measurements of the acidification capacity of treated cells and pH calibration curve. Bafilomycin A1 and chloroquine were used as positive de-acidification agents. All panels show representative Western blots from 3 experiments with triplicate measurements per experiment.

To study the possible mechanism underlying the hepatic steatosis, we again used HepG2 cells in which *TMEM199* or *CCDC115* was silenced using siRNA. Stimulation with OA (200 nmol) for 24 hours resulted in increased lipid accumulation in both *siTMEM199* and *siCCDC115* cell lines, but the patterns were different (Figure 4*A*). *TMEM199* silencing resulted in a more scattered lipid accumulation with significantly increased number of lipid droplets. In contrast, in *siCCDC115*-treated cells, lipid accumulation was observed in larger vesicles.

Because we did not observe a clearly increased expression of genes involved in lipid synthesis to explain the lipid accumulation, we next investigated whether reduced capacity of beta-oxidation could underly the lipid accumulation by investigating the respiratory capacity of the *siTMEM199*-treated cells using an Oroboros analyzer (Innsbruck, Austria). However, no changes were observed in oxygen consumption rates using either Palmitoyl-CoA or Palmitoyl-carnitine as a substrate (Figure 4*C*).

### Colocalization of Lipid Droplets With Lysosomes: Perturbed Autophagy and pH

To further characterize the lipid vesicles, we performed co-staining of neutral lipids and lysosomes using LipidTox (Thermo Fisher Scientific, Waltham, MA) and LysoTracker red DND-99 (Thermo Fisher Scientific), respectively. Interestingly, using Pearson’s correlation coefficient for LipidTox and LysoTracker red DND-99, we found significant colocalization of lipids in lysosomes in both models (*siTMEM199*: r = 0.76 ± 0.06, *P* < .0001; *siCCDC115*: r = 0.89 ± 0.02, *P* < .0001; control: r = 0.41 ± 0.02) (Figure 4*A*). Quantification of lipid droplet mean area and number of lipid droplets per cell revealed a significantly increased mean area in the *siTMEM199* (6.46 ± 0.84 μm^2^, *P* ≤ .01) and *siCCDC115* (11.97 ± 4.58 μm^2^, *P* = .0022) compared with control (3.54 ± 0.47 μm^2^) treated hepatocytes. We also found a significantly higher number of lipid droplets in the *siTMEM199* (49.31 ± 10.70, *P* < .01), but not in the *siCCDC115* (13.75 ± 2.56, not significant*)* treated cells when compared with controls (18.80 ± 7.80; Figure 4*B*).

Lysosomes and lysosomal degradation are integrated with autophagy, which led us to investigate autophagy parameters. Compared with their respective controls, the autophagy markers LC3 and P62 were significantly higher in *siTMEM199* and *siCCDC115* cells (Figure 4*D*) as well as in the livers of the hypomorphic *TMEM199* mouse model (Figure 4*E*), to the same extent as in controls cells treated with bafilomycin A1. Using LysoSensor (Thermo Fisher Scientific) to study whether lysosomal pH was affected, we observed significantly higher pH in lysosomes in both *siTMEM199* (pH 5.38) and *siCCDC115* HepG2 cells (pH 5.89) (Figure 4*F*). Together, these findings suggest an impaired autophagic flux due to reduced acidification of the autolysosome and may explain the increased lipid accumulation observed in the (auto)lysosome (Figure 4*A* and *C*).

## Discussion

Here, we report on the lipid abnormalities in plasma and liver observed in 2 recently found inborn errors of metabolism caused by mutations in 2 V-ATPase assembly factors, *TMEM199* and *CCDC115*. Patients are hypercholesterolemic, with most cholesterol sequestered in large VLDL particles. Consistent with this observation, we detected hypersecretion of apoB in loss-of-function hepatocyte models. Similarly, the steatosis observed in the patients was phenocopied in hepatocyte models by lipid droplet accumulation and increased lipid droplet–lysosome colocalization, as well as by the observation of hepatic steatosis in our hypomorphic mouse model of *TMEM199* deficiency.

In comparison with earlier reports on hyperlipidemia in other V-ATPase assembly defects,^4^, ^5^, ^6^ our study provides an in-depth characterization of the abnormal plasma lipids of these 2 V-ATPase assembly defects including apolipoproteins and FPLC profiles. We reveal that in both *TMEM199-* and *CCDC115* deficiency, elevated plasma cholesterol mostly sequesters in the VLDL fraction. Yet, these are likely not TG-rich lipoproteins, because plasma TG in patients was lower than in controls, suggesting a reduced ability of the liver to lipidate (V)LDL with TG before secretion. Compared with *VMA21* deficiency (25% increase in LDL-c) and *ATP6AP2* deficiency (on average 20% increase in LDL-c), the hyperlipidemia in *TMEM199* and *CCDC115* deficiency clearly was more pronounced (124% increase in LDL-c).

In search of a potential mechanistic explanation of the observed hypercholesterolemia in the *TMEM199* and *CCDC115* deficient patients, we next studied apoB secretion in 2 complementary cell models: HepG2 cells in which these factors had been silenced and induced pluripotent stem cell (iPSC)-derived hepatocytes from patients with *TMEM199* deficiency. Strikingly, in all these cell models we observed increased secretion of apoB, a novel finding for the V-ATPase assembly defects, not studied before in the other 3 V-ATPase assembly defects (ATP6AP1, ATP6AP2, or VMA21). Of note, we found no evidence that the hypercholesterolemia and hypersecretion of apoB were caused by differences in expression of genes involved in lipid metabolism, including the SREBPs and MTTP, in our cell and mouse models. Although it would have been desirable to characterize the VLDL fraction of the patient’s plasma in more depth by fractionation and by nuclear magnetic resonance spectroscopy, this was not feasible because of the very limited availability of plasma of these rare, often pediatric patients.

To study the plasma lipids and fatty liver phenotypes in a physiological context, we used CRISPR-Cas9–mediated genome editing to generate a knock-in mouse model that carries the Ala7Glu mutation in *TMEM199*, a mutation found in 1 of the 3 patients. This is a unique model, because no mouse models have been generated and characterized to date for any of the V-ATPase assembly defects (ie, ATP6AP1, ATP6AP2, and VMA21). Here, we show that the Ala7Glu mutation observed in patients results in loss of TMEM199 protein in our mouse model. In addition, plasma analysis of these mice indicated glycosylation defects comparable with patients with *TMEM199* deficiency showing hypogalactosylation of N-glycans. This may be secondary to disruption of the glycosylation enzymes in the Golgi apparatus, because zebrafish with defects in atp6ap2 have been shown to have dilated Golgi.^7^ Also, we observed hepatic steatosis in the livers of these mice, similar to the clinical steatosis in the patients, and we observed increased markers of autophagy, in line with our observations in the HepG2 cell models. Unlike in patients, *TMEM199*-deficient mice did not show a significant increase in VLDL-bound cholesterol or apoB. This is potentially due to the higher turnover of apoB-containing particles in murine plasma.^8^ The mice did exhibit a minor increase in TG content per VLDL-particle.

The increased number of lipid droplets described in liver biopsies of the patients with *TMEM199* and *CCDC115* deficiency combined with reports of disturbed autophagic flux in 2 other V-ATPase assembly defects^5^^,^^6^ led us to study lipid droplets and lysosomes in our HepG2 models of *TMEM199* and *CCDC115* deficiency. Lipid droplets are organelles with a core of neutral lipids—TG and cholesterol esters—surrounded by a phospholipid monolayer.^9^ Lipid droplets are catabolized by cytoplasmic lipases^10^ or via an autophagic route (also known as lipophagy).^11^ Indeed, the *TMEM199* and *CCDC115* deficient HepG2 models had an increased number and size of lipid droplets. Interestingly, our colocalization studies in both the *TMEM199* and *CCDC115* hepatocyte model showed disturbed interaction between lipid droplets and lysosomes. This is compatible with a disturbed autophagic flux of lipid droplets, and this was further supported by an increase in the autophagic markers LC3 and p62. In addition, with Lysosensor pH measurements we show that lysosomal pH is elevated in *siTMEM199* and *siCCDC115* HepG2 cells, likely because of misassembly of the V-ATPase complex. These pH values are above the optimal pH of LAL, which lies between 4 and 5,^12^ likely affecting its function, which in turn could contribute to the observed lipid droplet accumulation. Overall, this fits with a model that the disturbed V-ATPase assembly in *TMEM199* and *CCDC115* deficiency results in steatosis by defective lipid droplet turnover due to reduced organellar acidification in the lipid-droplet–autophagosome–lysosome axis.

These findings may also bear relevance for NAFLD, the increasingly prevalent form of hepatic steatosis and steatohepatitis, which is strongly associated with obesity and features of the metabolic syndrome, predominantly insulin resistance and diabetes mellitus type 2.^13^^,^^14^ Central to the development of NAFLD is hepatic lipid accumulation. This accumulation overwhelms the liver’s capacity for storage and mitochondrial oxidation of fatty acids, leading to lipotoxicity, a crucial step in the transition of simple steatosis to nonalcoholic steatohepatitis.^15^ The lysosomal autophagy of lipid droplets and the potential cross-talk between lysosome and autophagic regulators^16^ may be a central factor in protecting the liver from lipotoxicity. We postulate that deciphering regulators of lipophagy by studying V-ATPase assembly defects may help future targeting and drug development both for the common form of NAFLD and for these rare inborn errors of metabolism.

## Conclusion

We provide evidence that the hypercholesterolemia in *TMEM199* and *CCDC115* deficiency results from increased secretion of apoB-containing particles. Our data further suggest that the hepatic steatosis observed in these patients and in the mouse model is caused by aberrant interaction of lipid droplets and lysosomes. Together, these findings illustrate the importance of lipophagy, an understudied and largely untargeted mechanism that can potentially reduce lipotoxicity in fatty liver disease. Future studies aimed at the regulation of lipophagy may have therapeutic potential for these rare inborn errors of metabolism and possibly also for the common form of NAFLD.

## Methods

### Subjects

We collected plasma samples of 6 patients, 3 with homozygosity/compound heterozygosity for *TMEM199* mutations and 3 with homozygosity/compound heterozygosity for *CCDC115* mutations, whose clinical characteristics were reported earlier.^2^^,^^3^ In addition, we included 12 healthy, age- and gender-matched controls from the plasma biobank of the Amsterdam UMC, location AMC, The Netherlands. These were unaffected family members of patients with dyslipidemia without mutations in genes known to affect apoB and LDL-c (*APOB*, *LDLR,* and *PCSK9*) or in *TMEM199* or *CCDC115* from our lipid clinic with plasma stored in our blood bank.

### Plasma Lipids

We analyzed plasma lipids in venous blood samples collected after an overnight fast in EDTA-coated tubes. Plasma was isolated after centrifugation at 3000 RPM for 15 minutes at 4°C and stored at –80°C until further analysis. TC, LDL-c, HDL-c, TG, apoA-I, and apoB were measured using commercially available assays (DiaSys [Waterbury, CT] and WAKO) using a Selectra analyzer (Sopachem, The Netherlands). Cholesterol content in the main lipoprotein classes (VLDL, LDL, and HDL) was determined by using FPLC as described.^17^ The main system consisted of a PU-980 ternary pump with an LG-980-02 linear degasser and a UV-975 UV/VIS detector (Jasco, Tokyo, Japan). After injection of 30 μL plasma (1:1 diluted with TBS) the lipoproteins were separated using a Superose 6 Increase 10/30 column (GE Healthcare, Hoevelaken, The Netherlands). Because eluent TBS pH 7.4 was used at a flow rate of 0.31 mL/min, a second pump (PU-2080i Plus; Jasco) was used for either in-line cholesterol PAP or TG enzymatic substrate reagent (Sopachem) at a flow rate of 0.1 mL/min facilitating TC or TG detection. Commercially available plasma lipid standards (low, medium, and high) were used for generation of TC or TG calibration curves for the quantitative analysis (SKZL, The Netherlands) of the separated lipoprotein fractions. All calculations performed on the chromatograms were carried out with ChromNav chromatographic software, version 1.0 (Jasco).

### Cell Lines

HepG2 human hepatoma cells (ATCC-HB8065) were cultured in 75 cm^2^ flasks (Falcon) in complete medium (Dulbecco modified Eagle medium [DMEM] supplemented with 4.5 g glucose, 10% fetal bovine serum, and 1% penicillin/streptomycin; Gibco, Thermo Fisher Scientific). All cells were maintained in a 37°C humidified incubator supplemented with 5% CO_2_ atmosphere and maintained as described. For siRNA experiments, HepG2 cells were plated in 12-well plates at a density of 300,000 cells per mL. RNA silencing was done in HepG2 cells to mimic the *TMEM199* and *CCDC115* deficiency.

### Patient-Derived HLCs

Easily accessible differentiated cells, in this case fibroblasts, of 2 patients with *TMEM199* mutations and 2 age- and gender-matched controls, all obtained by skin biopsy at Radboud UMC, were reprogrammed to iPSCs and subsequently re-differentiated to HLCs (as described earlier).^18^^,^^19^ In short, patient-derived fibroblasts were reprogrammed with Cytotune reprogramming kit 2.0 by the iPSC core at University of Pennsylvania. First, 10 cell passages were cultivated on immortalized mouse embryonic fibroblasts as feeder cells in DMEM-F12 supplemented with 20% knockout replacement serum (Life Technologies), 4 ng/mL fibroblast growth factor**,** and incubated at 37°C with 5% O_2_/5% CO_2_**.** Cells were passaged when they reached sustainable colony size with Accutase (Innovative Cell Technology, San Diego, CA) according to manufacturer’s protocol. When the iPSCs reached appropriate confluency, they were transferred to Geltrex (Gibco) and cultivated in StemMACS iPSBrew (Miltenyi Biotec, Bergisch Gladbach, Germany). Passages were performed with StemMACS non-enzymatic passaging solution according to manufacturer’s instructions. After a minimum of 4 passages in iPSBrew, iPSCs were seeded 1 × 10^4^ cells per well in a 12-well plate coated with Geltrex. Induction of differentiation to definitive endoderm (days 1–5) was achieved with StemDiff (Stemcell Technologies, Vancouver, Canada) definitive endoderm kit according to manufacturer’s protocol. Further maturation of endoderm to mature induced hepatocytes (HLCs) was achieved according to Cai et al.^20^

### Gene Expression Analysis

Total RNA was isolated from the liver or cell lines using Trizol (Life Technologies) and reverse transcribed into cDNA using iSCRIPT (Bio-Rad, Hercules, CA). For qPCR, cDNA was amplified using Hi-ROX SensiMix SYBR green (Bioline, London, UK) or Taqman fast mix (Applied Biosystems, Foster City, CA) using the Quant Studio Real-Time qPCR (Applied Biosystems) with gene-specific primers (Table 2). Each sample was analyzed for relative expression by δδCT and normalized for 18S and 36b4.

**Table 2:** Primer Sequences Used for QPCR

Mouse cholesterol metabolism qPCR primers			
*SREBP2*		Forward	ACCTAGACCTCGCCAAAGGT
		Reverse	GCACGGATAAGCAGGTTTGT
*INSIG*		Forward	CTGGACGACGATGCCCAGGC
		Reverse	GTCACTGTGAGGCTTTTCCG
*HMGCS*		Forward	GCCGTGAACTGGGTCGAA
		Reverse	TCTGTTGTGAACCATGTGACTTC
*HMGCR*		Forward	TGGTGGGACCAACCTTCTAC
		Reverse	GCCATCACACGTGCCACATAC
*MTTP*		Forward	GATGTGGACGTTGTGTTACTGTGGAGGAATC
		Reverse	GAAGATGCTCTTCTCGCCTCTCTGTTGAC
*APOB*		Forward	CCCTGACAAGGATGAACCTAAATATATCCTG
		Reverse	TCTTGAATTCACGGTAACCTGAGTTGAGC
*LDLr*		Forward	GATGGCTATACCTACCCCTCAA
		Reverse	TGCTCATGCCACATCGTC
*PCSK9*		Forward	GAAGACCGCTCCCCTGAT
		Reverse	GCACCCTGGATGCTGGTA
Mouse de novo lipogenesis qPCR primers			
*LXR*		Reverse	CATCCTCTTCTCCCAGCA AG
		Forward	CATTACCAAGGCACTGTCCA
*SREBP1C*		Forward	GCAGACTCACTGCTGCTGAC
		Reverse	AGGTACTGTGGCCAAGATGG
*FASN*		Forward	GCTGCTGTTGGAAGTCAGC
		Reverse	AGTGTTCGTTCCTCGGAGTG
*CHREBP1*		Forward	GGCCTGGCTGGAACAGTA
		Reverse	CGAAGGGAATTCAGGACAGT
*SCD1*		Forward	ACTGGAGATCTCTTGGAGCA
		Reverse	CAGGTTTCCAAGCGCAGTTC
*CD36*		Forward	GATCGGAACTGTGGGCTCAT
		Reverse	GATCGGAACTGTGGGCTCAT
*ABCA1*		Forward	GGTTTGGAGATGGTTATACAATAGTTGT
		Reverse	GGTTTGGAGATGGTTATACAATAGTTGT
Human housekeeping qPCR primers			
*36b4*		Reverse	ACGGGTACAAACGAGTCCTG
		Forward	GCCTTGACCTTTTCAGCAAG
*18s*		Forward	GAGGGAGCCTGAGAAACGG
		Reverse	GTCGGGAGTGGGTAATTTGC
Mouse housekeeping qPCR primers			
*36b4*		Reverse	CCGGATGTGAGGCAGCAG
		Forward	GCTCCAAGCAGATGCAGCA
*18s*		Forward	CAC TTT TGG GGC CTT CGT G
		Reverse	GCA AAG GCC CAG AGA CTC ATT

### ApoB Secretion

ApoB secretion assays with or without OA stimulation were performed in the HepG2 cell model. Twenty-four hours after transfection the medium was changed to lipoprotein-deficient medium (DMEM supplemented with 4.5 g glucose, 10% lipoprotein-deficient serum, and 1% penicillin/streptomycin) (Life Technologies). After another 24 hours, either 0.3 mmol/L bovine serum albumin (BSA) conjugated OA (Sigma-Aldrich) in medium or fatty acid free BSA (Sigma-Aldrich) in lipoprotein-deficient medium was added to the wells. After 4 hours, the media were collected for apoB measurement, and cells were lysed with Trizol for RNA isolation for expression analysis or RIPA buffer with protease inhibitors (Roche) for analysis of protein levels. ApoB levels in the medium were measured with a specific enzyme-linked immunosorbent assay (as described by Kulozik et al^21^) and normalized for total cell protein using a BCA protein assay (Pierce, ThermoScientific). In HLCs, apoB secretion was assessed by ^35^S steady-state protein labelling and precipitation in methionine-free hepatocyte basal medium as described earlier.^22^^,^^23^

### Lipid Droplet and Lysosome Fluorescence Imaging of HepG2 Cells

For fluorescence microscopy, cells were grown on a coverslip to 50% confluency. For lysosomal staining, cells were incubated with 0.5 μmol/L Lysotracker Red DND-99 (Life Technologies; L7528) in full medium for 45 minutes before fixation. Cells were fixed with 4% PFA in phosphate-buffered saline for 10 minutes at room temperature. For determination of neutral lipids enclosed in cytosolic lipid droplets, cells were incubated with HCS LipidTOX Green neutral lipid stain (Life Technologies; H34475) according to manufacturer’s protocol. For imaging, cells were mounted with Vectaschield DAPI stain (Vector Laboratories, Burlingame, CA; H-1000-10) according to manufacturer’s protocol for nuclear staining. Cells were imaged at room temperature on a Zeiss (Jena, Germany) Observer Z1 brightfield microscope equipped with ApoTome 2 and a 63× (W) objective lens (APO DIC III numerical aperture 1.2) and acquired using Zeiss software Zen 2010. Laser lines used in this study were 405, 488, and 568 nm. Red/green/blue and greyscale images were further processed with Zeiss software Zen 2010. In addition, HepG2 cells and HLCs were used to assess cellular and surface LDL receptor protein expression levels with Western blot and flow cytometry and LDL uptake with Dylight labeled LDL particles in combination with flow cytometry.^24^

### Lysosomal pH Measurement With LysoSensor DND-160

HepG2 cells were seeded in a black walled glass bottom 96-well plate (Thermo Fisher Scientific; cat. #165305) and treated with siRNA as described. LysoSensor DND-160 staining was performed as described by Ma et al (2017)^25^ with some modifications. Modifications included LysoSensor DND-160 diluted to 5 μmol/L in full culture medium (DMEM with 10% fetal calf serum). Fluorescence intensity endpoint measurement was performed on a Synergy H2 microplate reader. Filter settings were excitation of Blue and Yellow 360/40 nm and emission 460/40 and 550/40, respectively.

### Western Blot Analysis

#### HepG2 cells

Cells were lysed with RIPA and harvested for Pierce BCA protein determination. Twenty-five micrograms protein was loaded on 14% Bis-Tris gel for electrophoresis and transferred to a polyvinylidene difluoride membrane. Membranes were blocked in 5% BSA, incubated with rabbit-anti-LC3 (Abcam, Cambridge, UK: ab192890) and rabbit-anti-p62 (R&D Systems, Minneapolis, MN: MAB8028) overnight at 4°C. Secondary horseradish peroxidase–conjugated donkey-anti-rabbit antibody was incubated for 1 hour at room temperature.

#### Mouse livers

Livers were homogenized in RIPA buffer and normalized for protein using the BCA assay. Protein samples were run on a 14% TRIS-Bis gel and transferred to a polyvinylidene difluoride membrane. These membranes were blocked with 5% skim milk in TBS-Tween 20 buffer and incubated with primary rabbit-anti-TMEM199 (ab121907) overnight at 4°C in 1% skim milk in TBS-Tween 20. Incubation with the secondary antibody was performed as above.

#### Mouse plasma

Western blots for apoB, PCSK9, and apoA1 were run by using 7 μL undiluted mouse plasma on a 4% Tris-Acetate gel. The proteins were in turn transferred to polyvinylidene difluoride membrane and blocked with 5% BSA in TBS-Tween 20. Overnight incubation at 4°C was done with primary rabbit anti-apoB (ab20737), PCSK9 (ERP7627), and apoA1 (EP1368Y). Incubation with the secondary antibody was performed as above.

### Mitochondrial Capacity

Oxygen consumption rates in HepG2 cells were measured by using a two-channel high-resolution Oroboros oxygraphy-2 k, in combination with the assay medium MiR05 pH 7.1 containing 110 mmol/L sucrose, 60 mmol/L potassium Lactobionate, 20 mmol/L taurine, 20 mmol/L HEPES, 0.5 mmol/L EGTA, 10 mmol/L KH2PO4, 3 mmol/L MgCl2, and 1 mg/mL BSA. Used substrates were (1) 25 μmol/L Palmitoyl-CoA in combination with 2 mmol/L L-carnitine and 2 mmol/L malate or (2) 25 μmol/L Palmitoyl-carnitine in combination with 2 mmol/L malate. Substrates were added after addition of 1 mmol/L of adenosine diphosphate to maximize the maximum substrate dependent coupled respiration flux (stage 3). Maximum uncoupled oxygen consumption (stage U) was determined after addition of 1.5 μmol/L carbonyl cyanide p-(trifluoro-methoxy) phenyl-hydrazone. Dithionite was used to eliminate oxygen out of the chamber and calibration of the oxygraph. Data acquisition and analysis were performed by using DatLab software version 7 (Oroboros). Oxygen flux was expressed as nmol/min/million cells.

### Tmem199-deficient Mice

As a *TMEM199* full knockout was embryonic lethal, C57BL/6J mouse embryos were gene-edited using CRISPR/Cas9 homology-directed repair knock-in using homologous alanine-7-glutamine (Ala7Glu) human-variant ssDNA oligos and inhibition of non-homologous end-joining using SCR7 (Xcess Bio, Chicago, IL) in the translational genome editing lab of Dr Raabe, Perelman School of Medicine, University of Pennsylvania. A guide RNA with the highest possible on-site cutting scores (using the MIT Crispr design website) found near the *TMEM199 A7* was designed. The sequence GAACGACTGGTTCGCGCTTT (adjacent PAM: GGG) was synthesized as ds DNA oligo with CACC 5' and AAAC 3' over hangs and inserted into the BbsI sites of plasmid pSpgRNA (Addgene#47108). A compatible DNA donor containing the allele TMEM199 E7 was also designed: Atccggcgcctgtgagcggaagtccgagtttgggatcacctgatcagagcgtgaggcagaaatggcgtcttccttgctt**gag**ggcgaacgactcgttagagctttgggtcctggcggagaactggaacgggagcagctcccccggaagctgcgtgcccagctcgaggctgctctgggaaa. The mutagenic codon for E7 (**gag**) is shown in bold, and several silent changes to prevent re-cutting by Cas9 after insertion of the DNA are underlined.

The in vitro activity of the above guide RNA on endogenous V6.5 mouse ES cell TMEM199 A7 was measured as follows: a mix of 0.5 μg of pSpgRNA plasmid DNA, 1 μg of donor DNA, 1 μg of pX330 Cas9 DNA plasmid (Addgene#42230), and 0.5 μg of a puromycin resistant plasmid for selection (Addgene#31937) was added to a well of a 6-well plate with V6.5 mouse ES cells in logarithmic growth phase, using lipofectamine 2000 as a transfection reagent according to the manufacturer’s recommendation. After selection with 2 μg/mL puromycin for 3 days the emerging clones were grown without puromycin another week, and 8 clones per genomic RNA were individually picked for further expansion in a 96-well plate. After growth until confluency for the majority of clones, genomic DNA was isolated with DNA lysis buffer containing 1 mg/mL proteinase K for 5 hours at 60°C. The lysate was carefully transferred to PCR tube strips. One volume of isopropanol was added to each tube, followed by thorough vortexing and spinning of the 96-well plate at 4000 rpm for 20 minutes at 4°C. The supernatant was carefully removed, and pellets were briefly exposed to air to remove traces of liquid. The pellets were dissolved in 50 μL DNA for 6 hours at 60°C with repeated vortexing. Targeting was analyzed for each clone by using the following PCR primers to amplify the *TMEM199 AE7* region: F1: 5'- GCCACACAACCAGCCATGTCCC -3', R1: 5'- CAATCTCGACTTCCTAAAAAAATTCAC -3'. PCR products were run on 2% agarose gel visualizing the number of clones that carried small deletions. Surprisingly, the above genomic RNA-donor DNA pair produced 3 homozygotic and 4 heterozygotic A7E mutations, with most heterozygotes carrying a deletion on the other allele.

Next, a T7 promoter was added to the genomic RNA by PCR using the T7 primer 5' ttaatacgactcactataggAACGACTGGTTCGCGCTTT and a universal reverse primer binding to the last 20 nucleotides of the constant genomic RNA region 5' aaaagcaccgactcggtgcc. After mini column purification the PCR product was transcribed using the message machine T7 kit (Thermo Fisher Scientific; #AM1344) according to manufacturer’s recommendations. The RNA was purified using the MEGA clear kit (Thermo Fisher Scientific; #AM1908). For generation of the *TMEM199 A7E* mouse the following injection mix was prepared: 120 ng/μL GR2 RNA, 150 ng/μL donor DNA, 300 ng/μL Cas9 protein (IDT, Newark, NJ), and 60 ng/μL Cas9 mRNA (Sigma-Aldrich). After mixing the components, 3 consecutive high-speed spins of 20 minutes at 4°C removed traces of glass fibers from the RNA mini column. The Penn Transgenic and Chimeric Mouse Facility injected the mix into the cytoplasm of 200 fertilized C57Bl6 zygotes and incubated them overnight in the presence of 50 μmol/L SCR7, a DNA ligase IV inhibitor (Xcess Bio). The best embryos were implanted into foster mice, resulting in 3 mice born heterozygous for the correct A7E mutation as assayed by tail DNA PCR using the same primers that were used for testing of the genomic RNAs. These mice were repeatedly bred to generate homozygous *TMEM199 A7E* mice.

Mouse plasma was used for plasma N-glycan profiling with MALDI-TOF mass spectrometry of permethylated glycans, as described earlier.^26^ FPLC profiling for cholesterol distribution across lipoprotein fractions was carried out as described.^17^ TG and cholesterol levels in FPLC fractions were measured by enzymatic assays and normalized to protein using the BCA assay.^27^

### Statistical Analyses

Data were compared between groups with Student *t* test and presented as means ± standard deviation or, when appropriate, tested with Mann-Whitney *U* test and presented as medians with interquartile ranges for nonparametric parameters. Categorical variables were tested with χ^2^ test. All statistical analyses were done using SPSS software (version 22.0; SPSS Inc, Chicago IL). Significance is indicated as ∗*P* < .05, ∗∗*P* < .01, ∗∗∗*P* < .001, ∗∗∗∗*P* < .0001.^28^
